# Programmed Death Ligand 1 Indicates Pre-Existing Adaptive Immune Response by Tumor-Infiltrating CD8^+^ T Cells in Non-Small Cell Lung Cancer

**DOI:** 10.3390/ijms20205138

**Published:** 2019-10-17

**Authors:** Yi-Ming Li, Jing-Min Yu, Zhen-Yu Liu, Hai-Jiao Yang, Juan Tang, Zhi-Nan Chen

**Affiliations:** 1 National Translational Science Center for Molecular Medicine, Xi’an 710032, China; sydlym@163.com (Y.-M.L.); yujingmin1226@163.com (J.-M.Y.); qqqzhenyu20052008@163.com (Z.-Y.L.); yhjjqq.love@163.com (H.-J.Y.); 2 Department of Cell Biology, School of Basic Medicine, Fourth Military Medical University, Xi’an 710032, China

**Keywords:** PD-L1, tumor infiltrating lymphocytes, IFN-γ, NSCLC

## Abstract

Aberrant expression of programmed death ligand 1 (PD-L1) on tumor cells impedes antitumor immunity and instigates immune evasion. The remarkable efficacy of immune checkpoint blockade has been confirmed in various solid tumors. However, the correlation between PD-L1 expression and host immunological landscape remains of considerable controversy in non-small cell lung cancer (NSCLC). In the present study, PD-L1 expression and CD8^+^ tumor-infiltrating lymphocyte (TIL) infiltration levels were determined by immunohistochemistry (IHC) in tumor sections of 138 NSCLC patients. The expression level of PD-L1 was positively correlated with the abundance of CD8 ^+^ TILs (*p* < 0.0001). Furthermore, no constitutive expression of PD-L1 was observed in the majority of six NSCLC cell lines detected by Western blot; but exposure to interferon-γ (IFN-γ), a primary cytokine secreted by activated CD8^+^ T cells, prominently increased PD-L1 expression. Notably, a significantly positive association was determined within PD-L1, CD8 and IFN-γ gene expression by qRT-PCR, which was corroborated by RNA-sequencing from TCGA lung cancer dataset. These findings demonstrate that PD-L1 expression indicates an adaptive immune resistance mechanism adopted by tumor cells in the aversion of immunogenic destruction by CD8^+^ TILs. Both higher expression of PD-L1 and infiltration of CD8^+^ TILs were correlated with superior prognosis (*p* = 0.044 for PD-L1; *p* = 0.002 for CD8). Moreover, Cox multivariate regression analysis showed that the combination of PD-L1 and CD8 were independent prognostic factors, which was more accurate in prediction of prognosis in NSCLC than individually. Finally, we found that IFN-γ induced the upregulation of PD-L1 in NSCLC cells, mainly through the JAK/STAT1 signaling pathway. In conclusion, PD-L1 expression is mainly induced by activated CD8^+^ TILs via IFN-γ in the immune milieu and indicates pre-existing adaptive immune response in NSCLC.

## 1. Introduction

Lung cancer remains to be the leading cause of cancer-related death worldwide, with non-small cell lung cancer (NSCLC) being the most common subtype accounting for 85% to 90% of all lung cancer [1]. Despite that the conventional therapy of NSCLC relying on surgical resection followed by systemic cytotoxic chemotherapy, radiation therapy and/or target therapy has evolved tremendously and rapidly over the past decade, the deteriorating existence of undruggable targets and drug resistance grievously limits the efficacy of treatment for NSCLC [2]. The recent advances in immunotherapeutic strategies, especially checkpoint blockade antibodies, show an unprecedented promise to improve clinical outcomes, may provide alternative therapeutic options in NSCLC, and have received widespread recognition [3]. Accumulative genomic alterations involved in the development and progression of NSCLC not only contribute to malignant phenotypes and behavior, but also exploit immune checkpoint molecules and pathways to escape from defensive destruction mediated by the immune system [4].

Dynamic interaction between tumor cells and immune cells in the tumor microenvironment is critical for carcinogenesis, and evasion of immunosurveillance plays a vital role in tumor progression. Indeed, increased tumor-infiltrating lymphocytes (TILs) have been consistently demonstrated to be associated with a better outcome in various human neoplasms [5]. CD8^+^ TILs have been proven to be independent of other prognostic variables and in correlation with preferable outcome in NSCLC [6]. However, numerous investigations to exploit adoptive immunotherapy, including tumor vaccines, cytokines and adoptive cell transfer have unsatisfyingly come to fruition in limitation, due to immune resistance and immune escape in overwhelming suppressive milieus or niches collectively constituted of Tregs, myeloid-derived suppressor cells (MDSCs), tumor-associated macrophages (TAMs), inhibitory cytokines, as well as a spectrum of repressive ligands and receptors in NSCLC.

Programmed death ligand 1 (PD-L1) is a key immune checkpoint molecule that has been implicated in tumor immune escape, and the immune-modulating antibodies against PD-L1 (Atezolizumab and Durvalumab) have already been approved for the treatment of NSCLC [7,8]. Binding of PD-L1 usurped by tumor to its receptor PD-1 on activated T cells, induces T cell anergy or exhaustion, attenuates T cell responses and facilitates tumor survival [9]. Previous studies have shown that patterns of PD-L1 expression and CD8^+^ TIL infiltration are associated with different pathological features and clinical outcomes in various cancers, such as gastric adenocarcinomas [10], colorectal cancer [11], hepatocellular carcinoma [12] and urothelial carcinoma [13]. Furthermore, objective benefit from PD-1/PD-L1 blockade therapy is shown in only a fraction of patients of NSCLC, indicating that development of strategies to effectively predict and optimize the treatment response of primary resistant tumors lacking PD-L1 expression and immune infiltration is needed urgently. Therefore, it is critical to focus on elucidating the mechanisms of regulating PD-L1 expression in NSCLC. Despite that intrinsic cellular changes in tumorigenesis may be the prime mover of constitutive expression of PD-L1 followed by immune evasion [14], increasing evidence indicates that upregulation of PD-L1 expression is inducible by infiltrative immune signals, including TILs, chemokines and pro-inflammatory/anti-inflammatory cytokines, as an adaptive response to anti-tumor immunity [15]. This co-existing pattern of constitutive and inducible expression of tumor PD-L1 derives from dynamic cross-talk between innate immune resistance and adaptive anti-tumor immunity. So far, the precise mechanisms of controlling tumor PD-L1 remain of controversy, due to limited data concerning the prevalence of PD-L1 expression in NSCLC. The present study was aimed to explicitly uncover the expression profile of PD-L1, as well as its correlation with local immune milieus in NSCLC. 

## 2. Results

### 2.1. Correlation between PD-L1 Expression, CD8^+^ TIL Infiltration and Clinical Characteristics

**Figure 1:**
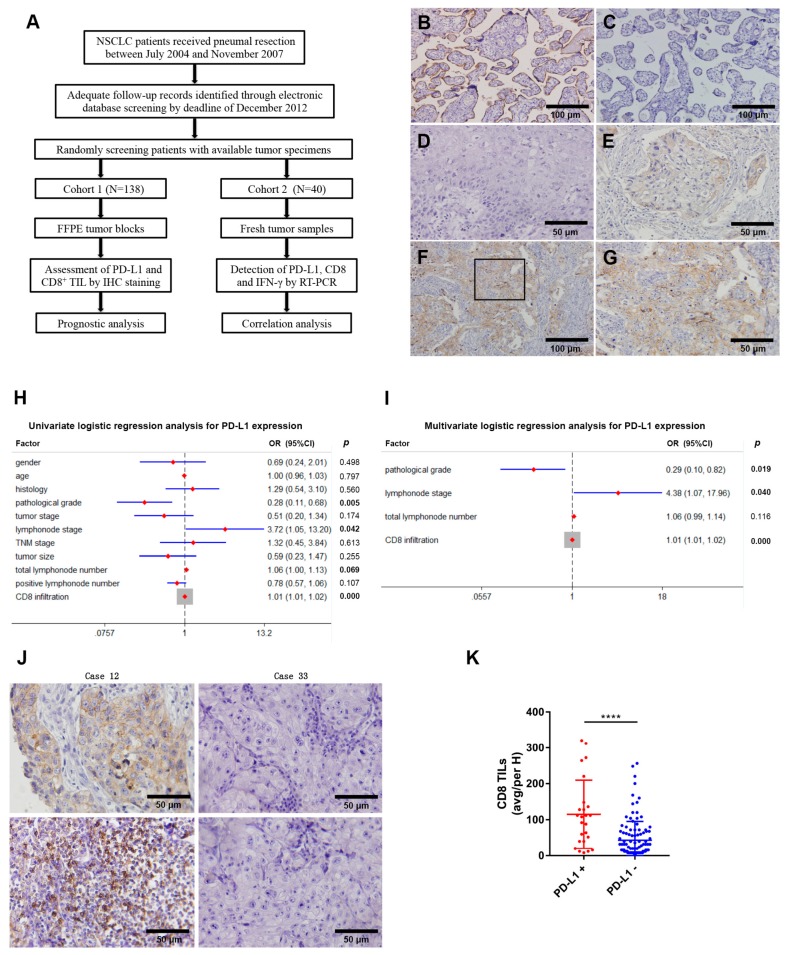
Correlation between PD-L1 expression, CD8^+^ TIL (tumor-infiltrating lymphocytes) infiltration and clinical characteristics. (**A**) Study design diagram. (**B**) A positive control of PD-L1 staining in human placenta tissue. (**C**) An isotype control for PD-L1 staining in human placenta tissue. (**D**) Negative PD-L1 expression on NSCLC tumor cells. (**E**) Weak PD-L1 expression on NSCLC tumor cells. (**F**) Strong PD-L1 expression on NSCLC tumor cells. (**G**) Original magnification of the boxed area shown in (**F**). (**H**) Univariate logistic regression analysis for PD-L1 expression. (**I**) Multivariate logistic regression analysis for PD-L1 expression. (**J**) Representative tumor sections accessed by IHC for PD-L1 expression on tumor cells and CD8^+^ TIL infiltration. PD-L1 positivity was defined by the presence of ≥5% of tumor cells; numbers of CD8^+^ TILs were manually counted in five randomly selected microscopic fields (200 magnification); and the mean was calculated. (**K**) Tumors were divided into two groups labeled by PD-L1^+^ and PD-L1^-^ followed by counting the number of CD8^+^ TILs. H, high magnification. **** *p* < 0.0001.

To evaluate PD-L1 expression, we consecutively enrolled 138 NSCLC patients with definite histopathological diagnosis, sufficient tumor specimens and adequate clinical data, as well as follow up information, whose baseline characteristics were concisely presented in Table 1. The median age of the patients analyzed was 61 years (range 20–84 years), and 103 (74.6%) were male. The most common histologic subtype was squamous cell carcinoma (*n* = 70 [50.7]%), and most patients were in TNM stage I (*n* = 65 [47.1]%) or II (*n* = 40 [29.0]%). The median follow-up is 53.3 months (range 1–96 months). Resection samples from a retrospective collection of NSCLC were randomly screened and divided into two cohorts independently (Figure 1A). 

To illuminate the biological prevalence of PD-L1 in NSCLC, we detected the expression of PD-L1 by immunohistochemistry in 138 NSCLC specimens. Overall, staining of PD-L1 showed a predominant cytoplasmic/membranous distribution and was present in only a subordinate fraction of all samples, among which 9 (6.5%) were strong PD-L1 positivity (Figure 1F) and 16 (11.6%) were weak PD-L1 positivity (Figure 1E), in comparison with negativity (defined as <5% PD-L1^+^ cells) (Figure 1D), positive control (Figure 1B) and isotype control (Figure 1C). Moreover, the expression level of PD-L1 was significantly higher in the tumor compartment than in stromal cells. The proportion of NSCLC cases showing PD-L1 expression by uniform screening in this cohort is consistent with previous studies and as similar as hepatocellular carcinoma [12], melanoma [16] and Merkel cell carcinoma [17]. 

Aiming to make further investigation of PD-L1 expression with tumor biology, we compared PD-L1 expression with clinical characteristics in NSCLC. To be specific, expression of PD-L1 was not significantly related to gender, age, histology, tumor stage, metastasis stage, TNM stage and tumor size, but significantly related to pathological grades, lymph node stage (*p* < 0.05, χ2 test [Table 2]). Univariate logistic regression analysis was performed for assessing the correlation of PD-L1 expression and clinical characteristics, which revealed that pathological grades (*p* = 0.005), lymph node stage (*p* = 0.042), total lymph node number (*p* = 0.069) and CD8^+^ TIL infiltrate (*p* < 0.0001) were statistically significant factors (Figure 1H). Furthermore, in a multivariate logistic regression analysis, including pathological grades, lymph node stage, total lymph node number and CD8^+^ TIL infiltrate, pathological grades (OR = 0.29; 95% confidence interval [CI]: 0.10–0.82; *p* = 0.019), lymph node stage (OR = 4.38; 95% confidence interval [CI]: 1.07–17.96; *p* = 0.040) and CD8^+^ TIL infiltrate (OR = 1.01; 95% confidence interval [CI]: 1.01–1.02; *p* < 0.0001) remained statistically significant (Figure 1I). It is evident that a continuous PD-L1/PD-1 interaction might be a mechanism employed by tumor cells to negatively regulate proliferation and cytotoxic response by CD8^+^ TILs and contributes to immune evasion in malignancy. 

To determine whether PD-L1 expression on tumor cells actually reflects a pre-existing immune cell-inflamed tumor microenvironment, we next accessed the association between PD-L1 expression and CD8^+^ TIL density by immunohistochemistry. Notably, PD-L1 expression was correlated with the density of CD8^+^ TILs in proportion (Figure 1J) and the number of CD8^+^ TILs was significantly higher in specimens with positive in comparison with negative PD-L1 expression (*p* < 0.0001, Figure 1K). Interestingly, one exception was of particular note in the 25 samples with PD-L1 positivity, which was characterized by high PD-L1 expression but with poor CD8^+^ TIL infiltration. The relative abundance of PD-L1^+^ tumor cells and CD8^+^ T cells was further analyzed by immune-fluorescence microscopy, which was consistent with the outcome of immunohistochemistry.

### 2.2. PD-L1, IFN-γ and CD8^+^ TILs in NSCLC

**Figure 2:**
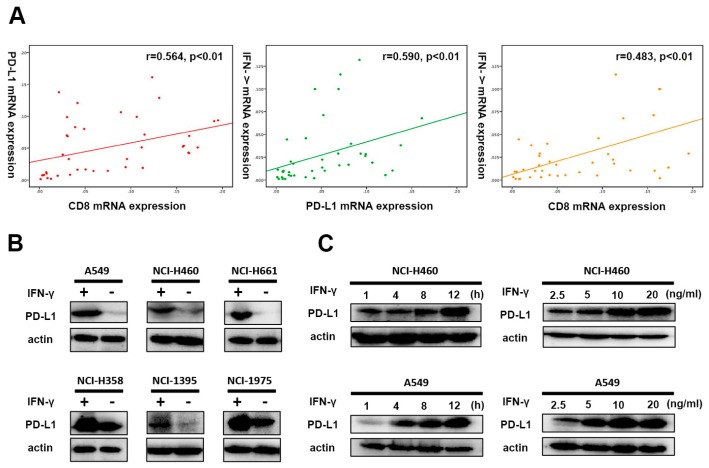
Correlation between PD-L1, CD8 and IFN-γ gene expression in NSCLC. (**A**) The expressions levels of PD-L1, CD8 and IFN-γ accessed by qRT-PCR in frozen NSCLC tumor tissues. Correlation analyses were performed for PD-L1 and CD8, PD-L1 and IFN-γ, CD8 and IFN-γ, respectively. GAPDH was utilized as an internal control. r: Spearman’s correlation coefficient. (**B**) Western blot detection of PD-L1 expression in NSCLC cell lines in the presence or absence of IFN-γ. (**C**) Sustained induction of PD-L1 by IFN-γ (20 ng/mL) was 1 h, 4 h, 8 h and 12 h, respectively. Concentrations of IFN-γ exposure range from 2.5 ng/mL to 20 ng/mL (2.5 ng/mL, 5 ng/mL, 10 ng/mL and 20 ng/mL, respectively). All experiments were conducted in triplicate.

To elucidate the potential mechanism behind the positive correlation between PD-L1 expression and CD8^+^ TILs in NSCLC, we randomly collected 40 surgically excised NSCLC specimens and quantitatively assessed the mRNA expression levels of PD-L1, CD8, and IFN-γ (a primary cytokine driving PD-L1 expression). The quantitative reverse transcription-polymerase chain reaction (qRT-PCR) determined a significantly positive correlation among the relative expression of PD-L1, CD8, and IFN-γ (r = 0.564, *p* < 0.01, for PD-L1 and CD8; r = 0.590, *p* < 0.01, for PD-L1 and IFN-γ; r = 0.483, *p* < 0.01, for CD8 and IFN-γ) (Figure 2A). It was also supported by the same trend of co-expression of PD-L1, CD8, and IFN-γ in peri-tumor in comparison with the corresponding tumor counterparts. 

In efforts to prove that upregulation of PD-L1 expression is inducible by CD8^+^ TILs via IFN-γ signaling, we treated 6 NSCLC cell lines for 24 h in the presence or absence of recombinant IFN-γ (20 ng/mL). No constitutive expression of PD-L1 was observed in the majority of these cell lines detected by Western blot. Treatment with IFN-γ stimulation prominently increased the expression level of PD-L1 (Figure 2B). Furthermore, upon IFN-γ treatment, there was a substantial increase in PD-L1 protein levels in a time-dependent manner, as well as a dosage-dependent manner (Figure 2C).

### 2.3. Association of PD-L1 and CD8^+^ TILs with Prognosis in NSCLC Patients

**Figure 3:**
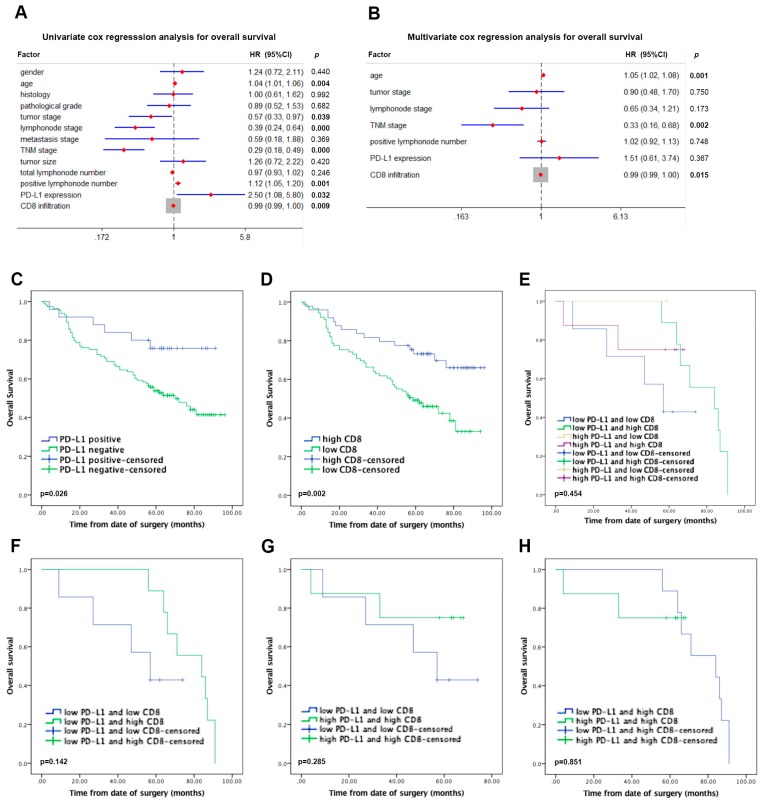
Association of PD-L1 and CD8^+^ TILs with prognosis in NSCLC patients. (**A**) Univariate cox regression analysis for overall survival. Bold numbers are statistically significant. (**B**) Multivariate cox regression analysis for overall survival. Bold numbers are statistically significant. (**C**) Kaplan-Meier curves for PD-L1 expression. (**D**) Kaplan-Meier curves for CD8^+^ TIL infiltration. (**E**) Kaplan-Meier curves for subgroups. (**F**) Kaplan-Meier curves for PD-L1^lo^CD8^lo^ and PD-L1^lo^CD8^hi^. (G) Kaplan-Meier curves for PD-L1^lo^CD8^lo^ and PD-L1^hi^CD8^hi^. (**H**) Kaplan-Meier curves for PD-L1^lo^CD8^hi^ and PD-L1^hi^CD8^hi^. *p*-values were calculated by log-rank test.

Overexpression of PD-L1 in tumor cells seems to be indicative of impaired anti-tumor immunity and escape surveillance by the immune system. However, PD-L1 remains of controversy as a predictive prognosis factor among previous reports [18,19,20]. Thus, we investigated the correlation of prognosis and PD-L1 expression, as well as CD8^+^ TILs in NSCLC patients and aimed to uncover whether the role of PD-L1 in tumorigenesis and progression had become paradoxical. After a median follow-up time of 53.3 months (range, 1–96 months), 65 of patients enrolled in the study had died. In the present study, results from 138 patients were available for analysis, including 18.1% cases with PD-L1 positivity and 81.9% cases with PD-L1 negativity. Univariate cox regression analysis, including gender, age, histology, pathological grade, tumor stage, lymph node stage, metastasis stage, TNM stage, tumor size, total lymph node number, positive lymph node number, PD-L1 expression, and CD8^+^ TIL infiltrate was performed for the assessment of their prognostic value, which revealed that age (*p* = 0.004), tumor stage (*p* = 0.039), lymph node stage (*p* < 0.0001), TNM stage (*p* < 0.000), positive lymph node number (*p* = 0.001), PD-L1 expression (*p* = 0.032) and CD8^+^ TIL infiltrate (*p* = 0.009) were significant (Figure 3A). Furthermore, multivariate cox regression analysis, including age, tumor stage, lymph node stage, TNM stage, and positive lymph node number was performed for the assessment of the independent prognostic value of PD-L1 expression and CD8^+^ TIL infiltrate. CD8^+^ TIL infiltrate was an independent predictor with improved overall survival (HR (hazard ratio) = 0.99; 95% confidence interval [CI]: 0.99–1.00; *p* = 0.015), but not PD-L1 expression (HR = 1.51; 95% confidence interval [CI]: 0.61–3.74; *p* = 0.367) (Figure 3B). 

The expression profile of PD-L1 was in positive correlation with favorable clinical outcome. For survival analysis, patients were divided into two groups, one with positive PD-L1 expression and one with negative PD-L1 expression. The Kaplan-Meier analysis highlighted significantly superior overall survival rates for the patients with PD-L1 positivity in comparison with those with PD-L1 negativity (*p* = 0.026; Figure 3C). Similarly, significantly increased overall survival was also observed in patients with high infiltration compared with low infiltration by CD8^+^ TILs (*p* = 0.002; Figure 3D). Localized infiltration of CD8^+^ TILs within tumor foci is associated with favorable patient survival, indicating that CD8^+^ TILs may induce immunogenic cell death and hold back tumor progression, which is consistent with previous studies [21,22]. Due to a significant positive correlation between PD-L1 and CD8 infiltrate in NSCLC, we further classified the patients into four subgroups: (1) PD-L1^lo^CD8^lo^; (2) PD-L1^lo^CD8^hi^; (3) PD-L1^hi^CD8^lo^; (4) PD-L1^hi^CD8^hi^. Kaplan-Meier analysis showed that no significant difference was observed among four subgroups (*p* = 0.454) (Figure 3E), as well as intercomparison (Figure 3F–H).

### 2.4. RNA-Sequencing from 1018 Whole Tissue Section Tumor Samples

**Figure 4:**
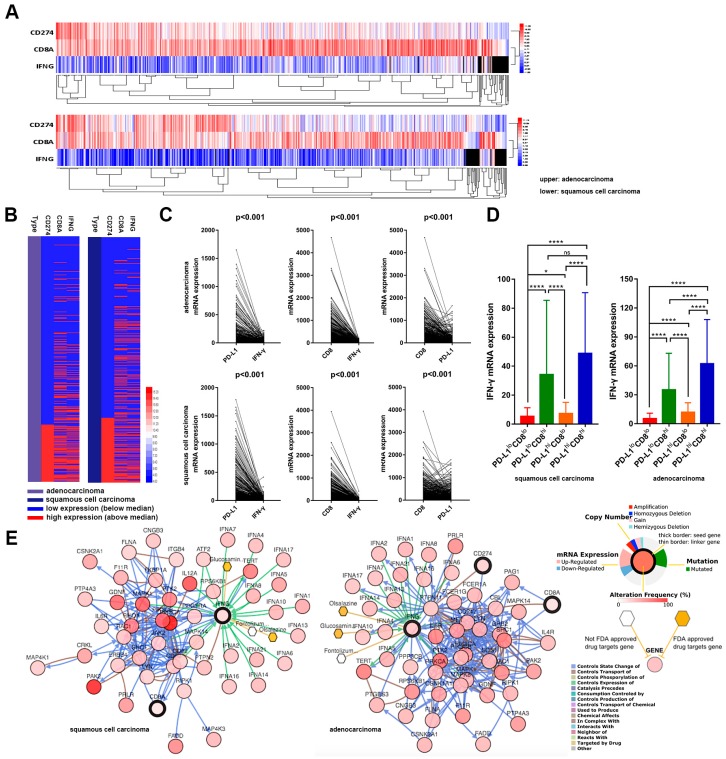
RNA-sequencing from 1018 whole tissue section tumor samples. (**A**) The cluster analysis of 1018 samples by RNA-sequencing. (**B**) The gene heat map of 1018 samples by RNA-sequencing. (**C**) Correlation analysis of PD-L1, CD8 and IFN-γ of 1018 samples. (**D**) IFN-γ abundance in four PD-L1 and CD8 immune infiltrate subgroups. * *p* < 0.05, ****p < 0.0001. (**E**) The network of related genes in the context of biological interactions with PD-L1, CD8, IFN-γ and promising biotargets for therapy of NSCLC.

To rule out possible bias induced by the limited tumor samples and/or the effect of marker heterogeneity, we further analyzed the mRNA levels of PD-L1, CD8, and IFN-γ acquired by RNA-sequencing from 1018 whole tissue section tumor samples from The Cancer Genome Atlas lung cancer datasets (lung squamous cell carcinoma, TCGA provisional, RNA Seq V2, 501 samples; lung adenocarcinoma, TCGA provisional, RNA Seq V2, 517 samples). The cluster analysis and gene heat map demonstrated that co-expression was of note among these three markers from 1018 samples by RNA-sequencing (Figure 4A,B). Furthermore, a restricted co-expression among these three markers was observed in both squamous cell carcinomas (*n* = 501; Pearson = 0.849, Spearman = 0.819, for CD8 and IFN-γ; Pearson = 0.285, Spearman = 0.403, for CD8 and PD-L1; Pearson = 0.232, Spearman = 0.375, for PD-L1 and IFN-γ) and adenocarcinomas (*n* = 517; Pearson = 0.737, Spearman = 0.793, for CD8 and IFN-γ; Pearson = 0.344, Spearman = 0.555, for PD-L1 and IFN-γ; Pearson = 0.290, Spearman = 0.537, for CD8 and PD-L1), and remained statistically significant (*p* < 0.001) (Figure 4C). Subgroup analysis also revealed distinct IFN-γ abundance in different PD-L1, and CD8 immune infiltrates (Figure 4D). The gene network showed related genes in the context of biological interactions with PD-L1, CD8 and IFN-γ and revealed promising biotargets for therapy of NSCLC (Figure 4E). 

### 2.5. IFN-γ-Induced PD-L1 Upregulation is Dependent on the JAK/STAT1 Signaling Pathway

**Figure 5:**
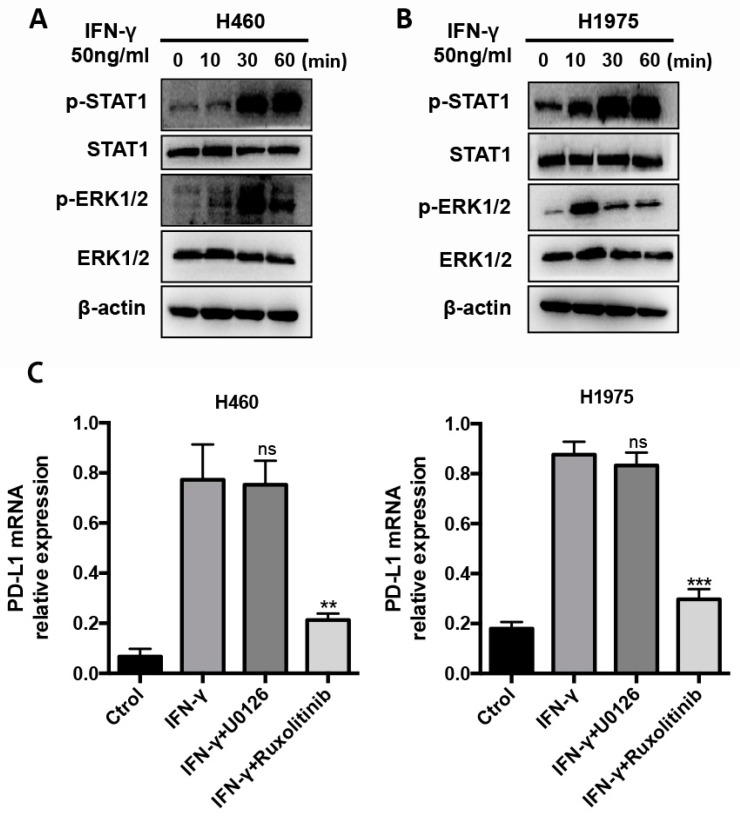
IFN-γ-induced PD-L1 upregulation is dependent on the JAK/STAT1 signaling pathway. (**A**) Activation of STAT1 and ERK1/2 signalings was analyzed by Western blot in H460 cells treated with 50 ng/mL of IFN-γ for 0, 10, 30 and 60 min. The same samples were run on parallel gels to obtain the bands of phosphorylated protein and total protein. (**B**) Activation of STAT1 and ERK1/2 signalings was analyzed by Western blot in H1975 cells treated with 50 ng/mL of IFN-γ for 0, 10, 30 and 60 min. The same samples were run on parallel gels to obtain the bands of phosphorylated protein and total protein. (**C**) PD-L1 expression was detected by quantitative RT-PCR in H460 and H1975 cells after incubation with IFN-γ in the presence or absence of signaling inhibitors, U0126 and Ruxolitinib. Ctrol, Control. **p < 0.01, ***p < 0.001.

The molecular pathways orchestrating the cellular response to IFN-γ stimulation are cell type- and gene-specific, mainly, including JAK/STAT1, JNK, ERK1/2, PI3K/AKT and NF-κB pathways, which were activated by engagement of IFN-γ receptors by IFN-γ. To further investigate the major molecular pathways responsible for IFN-γ-induced PD-L1 upregulation, we performed signaling activation experiments in NSCLC cells. Given that EGFR mutation or ALK fusion may alter the PD-L1 expression, we used two NSCLC cell lines, including NCI-H460 cells and NCI-H1975 cells, the latter characterized by EGFR-T790 mutation. We found that IFN-γ treatment-induced the activation of STAT1 and ERK1/2 signaling pathways in both H460 and H1975 cells. The expression level of phospho-STAT1 (*p*-STAT1) in IFN-γ-stimulated NSCLC cells was significantly higher than that in the non-stimulated NSCLC cells (Figure 5A). In addition, the expression level of phospho-ERK1/2 (*p*-ERK1/2) in IFN-γ-stimulated NSCLC cells was significantly higher than that in the non-stimulated NSCLC cells (Figure 5B). Then, we used signaling pathway inhibitors U0126 and Ruxolitinib to inhibit ERK1/2 and JAK/STAT1 signaling pathways, respectively. We found that the JAK inhibitor significantly reduced the induction of PD-L1 expression in comparison to the ERK1/2 signaling inhibitor (Figure 5C). These results converge to suggest that IFN-γ induced the upregulation of PD-L1 in NSCLC cells, mainly through the JAK/STAT1 signaling pathway.

## 3. Discussion

Mechanistically, it has been well documented that PD-L1 expressed on tumor cells would facilitate tumor immune tolerance and evasion of the host by interacting with its receptor PD-1 on T cells and leading to T cell inactivation or exhaustion in the tumor microenvironment [23]. PD-L1 has been traditionally considered as a negative co-stimulatory molecule promoted constitutively by oncogenic driver mutations and indicates defective adaptive immune response in many solid tumors [24]. Following this rationale, the overexpression of PD-L1 by tumor cells behooves to correlate with poorer prognosis [25]. However, PD-L1 expression on tumor cells could be attributed to IFN-γ production by TILs, which is in association with powerful anti-tumor immunity and favorable prognosis in theory. Actually, the diversity remains in the outcome obtained from different studies investigating whether PD-L1 could be recognized as an effective biomarker of prognosis. Recent clinical trials revealed different predictive values for PD-L1 expression in NSCLC, which indicates that high PD-L1 expression is an unfavorable prognostic factor in most of the studies [26,27,28]. Nevertheless, no direct evidence was observed in favor of the correlation between PD-L1 overexpression with defective immune response in previous studies. Based on previous studies, the three main contributions of our study are summarized as follows. Firstly, we quantitatively assessed PD-L1, CD8 and IFN-γ gene expression by using RT-qPCR in NSCLC fresh tumor samples as a validation cohort, apart from common semi-quantitative immunohistochemistry. Secondly, we innovatively performed RNA-sequence to evaluate PD-L1, CD8 and IFN-γ gene expression from a large-scale population, which contains 1018 whole tissue section tumor samples. Thirdly, we further revealed related genes in the context of biological interactions with PD-L1, CD8 and IFN-γ by bioinformatics in TCGA databases. Amazingly, our investigation shows that the expression profile of PD-L1 was in proportion to the infiltration density of CD8^+^ TILs and achieved better prognosis within the follow-up. This discovery, in which overexpression of an immunosuppressive molecule was closely linked to improved prognosis, endorse the perspective that PD-L1 indicates an adaptive immune resistance mechanism adopted by tumor cells in the aversion of immunogenic destruction by TILs [15,16,28,29,30]. 

Several aspects concerning the predictive value of prognosis by PD-L1 should be worthy of note. Firstly, no standard criteria for the cut-offs on PD-L1 overexpression have been proffered, which results in various definition thresholds having been proposed and adopted. Secondly, tissue microarrays utilized in most of the researches may bias screening estimates of the expression profile of PD-L1, due to the prevalence of low PD-L1 expression and finite pathological specimen. On the contrary, whole tissue sections usually result in a higher prevalence of PD-L1 expression. Moreover, while PD-L1 has been recognized as a potential diagnostic and therapeutic biomarker in the clinic, discrepant methodologies characterized by different anti-PD-L1 antibodies applied for IHC staining may also contribute to the inconformity within the previous studies (such as ab205921, abcam; E1L3N, Cell Signaling Technology; etc.) [30,31,32,33,34]. The results in the present study coincide with the outcome by noncommercial PD-L1 antibody clone 5H1, which has been widely accepted in diagnostic pathology [17,35]. In addition, preoperative radiochemotherapy may induce overexpression of PD-L1 mediated by activated TILs via the explosive release of tumor-associated molecules (TAMs), which indicates that identification or development of strict inclusion criteria should be put on the agenda. Most importantly, it is worthy of further investigating the nature of dynamic and complex immune responses by stimulatory and inhibitory signals in the tumor immune microenvironment.

IFN-γ produced by activated TILs has been found to be precedent and indispensable for PD-L1 expression. Within the melanoma tumor microenvironment, induction of PD-L1 and IDO on tumor cells depends on IFN-γ produced by activated CD8^+^ TILs [15]. Furthermore, a randomized controlled trial showed that favorable overall survival rates were observed in patients with high expression of T-effector-IFN-γ-associated genes, which was carried out in an NSCLC cohort pretreated by docetaxel or atezolizumab [36]. These studies support our hypothesis that therapeutic benefit deriving from immune checkpoint blockade is prominent in malignancy with pre-existing immunity. In order to reveal the latent mechanism underlying the correlation between PD-L1 induction and CD8^+^ TIL infiltration, the mRNA levels of PD-L1, CD8 and IFN-γ were accessed by RT-PCR in 40 NSCLC specimens, showing that *IFN-γ* is significantly associated with *PD-L1* and *CD8* gene expression. Next, we showed that PD-L1 expression was induced or augmented by exposure to IFN-γ in NSCLC cell lines. Thus, it is likely that PD-L1 overexpression on tumor cells resulted from the release of driving cytokines secreted from the TILs in the tumor microenvironment, indicating that immunotherapies aimed with checkpoint blockade or signal disengagement might be of extraordinary efficacy in tumors with abundant TIL infiltration during the negative feedback stage of smoldering immune response, despite the complexity and dynamics of immune regulation networks in vivo [37]. 

Our in vitro results demonstrate that NSCLC cells do not normally express PD-L1 or express very little of PD-L1 by Western blot. However, we reported that PD-L1 positivity in NSCLC samples is around 20% by immunohistochemistry and in a larger cohort, including 982 patients, 314 (32.0%) specimens were positive for PD-L1 expression [38]. Hence, we hypothesized that PD-L1 on tumor cells is not expressed constitutively, but expressed in response to IFN-γ exposure in the tumor microenvironment. Furthermore, we verified that PD-L1 expression is time and dosage-dependent, while the level of PD-L1 reaches its peak within a relatively short period that is regarded as “transient” as well [39]. We also found that JAK/STAT1 pathway is activated in IFN-γ-induced PD-L1 expression in NSCLC cells. Blockade of JAK/STAT1 pathway abrogates the PD-L1 induction by IFN-γ. In addition to extrinsic mechanisms, it is of note that in the 25 PD-L1 positive specimens, one sample was distinctive from others by poor infiltration of CD8^+^ T cell infiltration, which implies that intrinsic signals triggered by carcinogenesis may also get involved in the regulation of PD-L1 expression. It is reported that PD-L1 expression was regulated by intracellular oncogenic activation of the AKT-mTOR pathway in syngeneic and genetically engineered mouse models of NSCLC [40]. Besides, loss of tumor suppressor PTEN function also increased PD-L1 expression and facilitated immune resistance in glioma [41]. In a mouse model of pancreatitis, autophagy repressed by deletion of *Atg5* dysregulated TBK1 signaling and upregulated PD-L1 [42]. In brief, both extrinsic and intrinsic mechanisms may drive independently or synergistically to regulate the expression of PD-L1 in the tumor microenvironment.

Collectively, these findings contribute to investigating the praxiology and genomics of both the tumor and the host immune landscape, which play critical roles in discovering and validating potential biomarkers for malignancy. Upon interaction between PD-L1 on tumor cells and PD-1 on immune cells, inhibitory signals are triggered and result in inactivation or even apoptosis of TILs, which protects tumor cells from immunogenic death by host immunity [43]. An increasing number of studies have shown that tumor PD-L1 overexpression reflects an active tumor immune microenvironment and may be the premise behind the aforementioned PD-L1/PD-1 inhibitor therapy in which release of the brake indicates as more efficient than the reestablishment of an existing TIL tumoricidal capability de novo [44]. Nivolumab, a PD-1-targeted mAb, has shown promising clinical effects in therapy of advanced metastatic NSCLC, unresectable or metastatic melanoma and advanced renal cell carcinoma [45,46,47]. Atezolizumab, a PD-L1-targeted mAb, was recently approved for therapy of locally advanced or metastatic urothelial malignancy with cisplatin ineligibility and showed favorable outcome for advanced NSCLC [48,49]. Nevertheless, the effectual clinical benefits of checkpoint blockade are hampered by a high rate of primary drug-resistance, where only a small proportion of recipients respond to the therapy, suggesting that larvaceous reversal of host immunity by PD-L1/PD-1 blockade might hinge on the abundance of CD8^+^ TILs infiltrated in the tumor microenvironment. Therefore, developing novel strategies to stratify patients better, based on individual response to PD-L1/PD-1 blockade is much warranted. Terri et al. identified a gene expression profile (GEP) that correlates with clinical response to Pembrolizimab, and found that the presence of this T cell-inflamed GEP was of indicative of response to immune therapy [50].

There are also some limitations in this work. Firstly, selection bias and allocation concealment were inevitable in this retrospective study, which calls for a multi-center study with a larger cohort. Secondly, PD-L1 expressed on immune cells might not be distinguished definitely from that on tumor cells merely by IHC, for which flow cytometry may contribute to the definition of PD-L1 expression in various cell types in the tumor microenvironment. Last, but not least, the relatively longer follow-up may facilitate for collecting more precise and robust clinical data. 

In conclusion, the presented findings of this study contribute to uncovering the characterization of the complicated features inherent with tumor immune microenvironment in NSCLC, which demonstrates that PD-L1 expression is mainly induced by activated CD8^+^ TILs via IFN-γ in the immune milieu and indicates pre-existing adaptive immune response for favorable prognosis in NSCLC. Our findings proffer a fundamental rationale for further investigation into novel therapeutic avenues for refining the coverage of checkpoint blockade therapy in NSCLC.

## 4. Materials and Methods

### 4.1. Cell Culture and Cytokine Treatment

Six NSCLC cell lines purchased from American Type Culture Collection (ATCC) included A549 (No. CCL-185), NCI-H460 (No. HTB-177), NCI-H661 (No. HTB-183), NCI-H358 (No. CRL-5807), NCI-H1395 (No. CRL-5868) and NCI-H1975 (No. CRL-5908). Cells were plated in 100 mm^2^ petri dish, cultured at 37 °C, 5% CO_2_ in RPMI 1640 supplemented with 10% fetal bovine serum and 1% penicillin-streptomycin and allowed to reach 70–80% confluence. As for cytokine treatment, cells were washed 3x with PBS and incubated for 24 h in serum-free RPMI 1640. Cells were treated with recombinant human interferon-γ (IFN-γ, #300-02, Peprotech) in serum-free RPMI 1640 and harvested for protein isolation. Controls included both normal serum concentration group and serum-starved group. 

### 4.2. Patients, Cohorts and Specimens

All patients enrolled in the study were diagnosed with NSCLC and underwent pneumal surgical resection between 2004 and 2007. The study design was approved by the Ethics Committee, and informed consent was acquired from all patients. Histopathological sections of each patient by HE staining were reviewed by two pathologists to evaluate the grades and TNM stages individually. The TNM stages were assessed based on the American Joint Committee on Cancer (AJCC) TNM classification (the seventh edition) for NSCLC. Tumor differentiation grading was classified by the Edmondson grading system. Eligible patients in this study were those with histopathological confirmation, sufficient samples for IHC staining and qRT-PCR, and complete clinical and outcome statistics. Through screening electronic database by follow-up deadline of December 2012, we retrospectively collected those patients with adequate follow-up information. Previous studies suggested that PD-L1 expression status might vary across different histologic types or stages of NSCLC; thus, we randomly screened patients with available tumor specimens and collected samples from patients with different stages and histologic types so as to be in both randomness and representativeness. Cohort 1 was composed of 138 patients with available formalin-fixed paraffin-embedded (FFPE) tumor blocks to assess the expression of PD-L1 and CD8^+^ TILs by IHC staining for prognostic analysis. Overall survival (OS) rates were calculated from the date of surgery to death or until the last follow-up (censored). Cohort 2 was composed of 40 patients with frozen tumor samples to detect PD-L1, CD8 and IFN-γ by RT-PCR for correlation analysis. 

### 4.3. Immunohistochemistry (IHC)

4 µm thick sections were deparaffinized, followed by antigen retrieval with 10 μmol/L citrate buffer at pH6.0. Endogenous peroxidase activity was blocked with methanol containing 3% hydrogen peroxide for 15 min. After serum block, sections were incubated with anti-PD-L1 (Clone E1L3N, #13684, Cell Signaling Technology) and anti-CD8 (#ET1606-31, Hua An Co., Hangzhou, China) at 4 °C overnight. Following incubation, immunoperoxidase staining was carried out by a streptavidin-peroxidase kit (Zhongshan Jinqiao Co., Beijing, China) and treated with 3,3′-diaminobenzidine (Zhongshan Jinqiao Co.) to visualize the target proteins. Hematoxylin was utilized to counterstain the nuclei. Isotype control and negative control were used to evaluate the specificity of all antibodies. 

### 4.4. Assessment of Immunostaining Parameters

All immunohistochemical slides were evaluated by two independent pathologists blinded to the clinical data of the specimens. PD-L1 positivity was defined by the existence of ≥5% of tumor cells, which is in accordance with the threshold of a previous large-scale phase I clinical trial accessing the safety, activity and immune correlates of an anti-PD-1 antibody [51]. Numbers of CD8^+^ TILs were manually counted in five randomly selected areas (200 magnification); and the mean was calculated. The median values were utilized as the cut-off in the following analyses.

### 4.5. RNA Extraction and qRT-PCR Analysis 

Total RNA was extracted from specimen tissues by utilization of Total RNA Kit II (Omega Bio-tek, Norcross, GA, USA) following manufacturer’s instructions, and then reversely transcribed to cDNA by use of the PrimeScript RT Reagent Kit (TaKaRa, Ohtsu, Japan). The SYBR Premix Ex Taq II Kit (TaKaRa) was used in qRT-PCR. The sequences of PCR primers were listed as follows.

Human PD-L1-F: 5′-TGGCATTTGCTGAACGCATTT-3′,
Human PD-L1-R: 5′-TGCAGCCAGGTCTAATTGTTTT-3′;
Human CD8-F: 5′-ATGGCCTTACCAGTGACCG-3′,
Human CD8-R: 5′-AGGTTCCAGGTCCGATCCAG-3′;
Human IFN-γ-F: 5′-TCGGTAACTGACTTGAATGTCCA-3′,
Human IFN-γ-R: 5′-TCGCTTCCCTGTTTTAGCTGC-3′;
Human GAPDH-F: 5′-CTCCTCCTGTTCGACAGTCAGC-3′,
Human GAPDH-R: 5′-CCCAATACGACCAAATCCGTT-3′.

### 4.6. RNA-Sequencing from The Cancer Genome Atlas (TCGA)

To explore the correlation between PD-L1, CD8 and IFN-γ expression, two RNA-sequencing large-scale NSCLC genomics data sets from The Cancer Genome Atlas (TCGA) were employed and analyzed by the cBioPortal for Cancer Genomics [52,53]. The two data sets were lung squamous cell carcinoma, TCGA provisional, RNA Seq V2, 501 samples; lung adenocarcinoma, TCGA provisional, RNA Seq V2, 517 samples.

### 4.7. Western Blot Analysis

Human NSCLC cancer cells were harvested and then treated with RIPA lysis buffer containing phenylmethylsulfonyl floride, protease inhibitors and phosphatase inhibitors (KeyGen BioTech, Nanjing, China). Lysates were incubated on ice for 30 min, centrifuged (14,000 *rpm*, 20 min, 4 °C) and then supernatants were subjected to the subsequent SDS-PAGE. Protein amounts were quantified by the BCA Protein Assay Kit (Thermo Fisher Scientific, Waltham, MA, USA). Protein samples were boiled for 10 min and then loaded to 12% SDS-PAGE gel by equal amounts. Gels were transferred to PVDF membranes (Millipore, Billerica, MA, USA). After blockade in 5% non-fat milk in TBST (50 mM Tris/HCl, pH 7.4, 150 mM NaCl, 0.1% Tween-20) for 1 h, the membrane was incubated with the designated primary antibodies (anti-PD-L1, Clone E1L3N, #13684, Cell Signaling Technology, dilution 1:800; anti-β-actin, #M1210-2, Hua An Co., dilution 1:2000) at 4 °C overnight. The images were developed after incubation with the secondary antibodies (goat anti-rabbit IgG (H + L) antibody, #31460, Thermo Fisher Scientific, dilution 1:5000; goat anti-mouse IgG (H + L) antibody, #31430, Thermo Fisher Scientific, dilution 1:5000) at room temperature for 1 h. Bands were visualized by Chemidoc Touch (Bio-Rad, Hercules, CA, USA).

### 4.8. Statistical Analysis

The statistical analyses were performed by Stata 14.0 (StataCorp.), SPSS 23.0 (SPSS, Inc.) and GraphPad Prism 7 (GraphPad Software, Inc.). The association between immune parameters and clinicopathological features was analyzed by chi-square test or Fisher’s exact test (*p* value (two-sided) <0.10 was regarded as statistically significant). Spearman’s rank correlation was employed to analyze the association of PD-L1, CD8 and IFN-γ mRNA expression. Comparisons of CD8^+^ TIL amounts were conducted by Student’s t test and ANOVA. Overall survival was calculated using Kaplan-Meier analysis and compared by the log-rank test. Survival analysis was conducted by multivariate Cox regression models (*p* value (two-sided) <0.10 was regarded as statistically significant). 

## Figures and Tables

**Table 1:** General clinicopathological features of non-small cell lung cancer (NSCLC) patients.

Variables	Results
Gender (female/male)	35/103
Median (range) age (year)	61 (20–84)
Tumor size (small/large)	101/37
Grade (I + I-II + II /II-III + III)	99/39
T stage (T1/T2/T3/T4)	31/77/21/9
N stage (N0/N1/N2/Nx)	76/21/17/24
M stage (M0/M1)	134/4
TNM Stage (I/II/III/IV)	65/40/29/4
Histology (SCC/ADC)	70/68
Driver mutations (EGFR/ALK/Both)	59/6/2
Treatments (CCRT with platinum/CCRT)	90/48
Median (range) follow-up (months)	53.3 (1–96)
Status (alive/dead)	73/65

Small, ≤5 cm; Large, >5 cm.

**Table 2:** PD-L1 expression in different clinicopathological features of NSCLC patients.

				PD-L1 Expression						
				Strong Positive		Weak Positive		Negative Positive		
Subgroup	Variables	No.	%	No.	%	No.	%	No.	%	*p* Value ^α^
Overall Gender		138	100	9	6.5	16	11.6	113	81.9	0.591
	F	35	25.4	1	2.9	4	11.4	30	85.6	
	M	103	74.6	8	7.8	12	11.7	83	80.6	
Age										0.839
	<65	79	57.2	6	7.6	9	11.4	64	81.0	
	≥65	59	42.8	3	5.1	7	11.9	49	83.1	
Histology										0.843
	SCC	70	50.7	5	7.1	9	12.9	56	80.0	
	ADC	68	49.3	4	5.9	7	10.3	57	83.8	
Grades										<0.001
	I + I-II + II	99	71.7	2	2.0	10	10.1	87	87.9	
	II-III + III	39	28.3	7	17.9	6	15.4	26	66.7	
Tumor (AJCC)										0.146
	T1	31	22.5	1	3.2	5	16.1	25	80.6	
	T2	77	55.8	3	3.9	8	10.4	66	85.7	
	T3	21	15.2	4	19.0	1	4.8	16	76.2	
	T4	9	6.5	1	11.1	2	22.2	6	66.6	
Node (AJCC)										0.024
	N0	76	55.1	5	6.6	13	17.1	58	76.3	
	N1	21	15.2	4	19.0	0	0.0	17	81.0	
	N2	17	12.3	0	0.0	0	0.0	17	100.0	
	Nx	24	17.4	0	0.0	3	12.5	21	87.5	
Metastasis stage (AJCC)										1.000
	M0	134	97.1	9	6.7	16	11.9	109	81.3	
	M1	4	2.9	0	0.0	0	0.0	4	100.0	
TNM stage (AJCC)										0.257
	I	65	47.1	1	1.5	10	15.4	54	83.1	
	II	40	29	5	12.5	4	10.0	31	77.5	
	III	29	21	3	10.3	2	6.9	24	82.8	
	IV	4	2.9	0	0.0	0	0.0	4	100.0	
Tumor size										0.370
	Small	101	63	8	7.9	13	12.9	80	79.2	
	Large	37	37	1	2.7	3	8.1	33	89.2	

**^α^** Determined with χ^2^ test. F, female; M, male; SCC, squamous cell carcinoma; ADC, adenocarcinoma.
